# Supplementary material for: Comparative Analysis of rRNA Removal Methods for RNA-Seq Differential Expression in Halophilic Archaea
Source: Biomolecules. 2022 May 10;12(5):682. doi: 10.3390/biom12050682 (PMC9138242; doi:10.3390/biom12050682)
Supplement: Supplementary file 1 [file biomolecules-12-00682-s001.zip › biomolecules-1705790-supplementary/Figure S1.pdf]

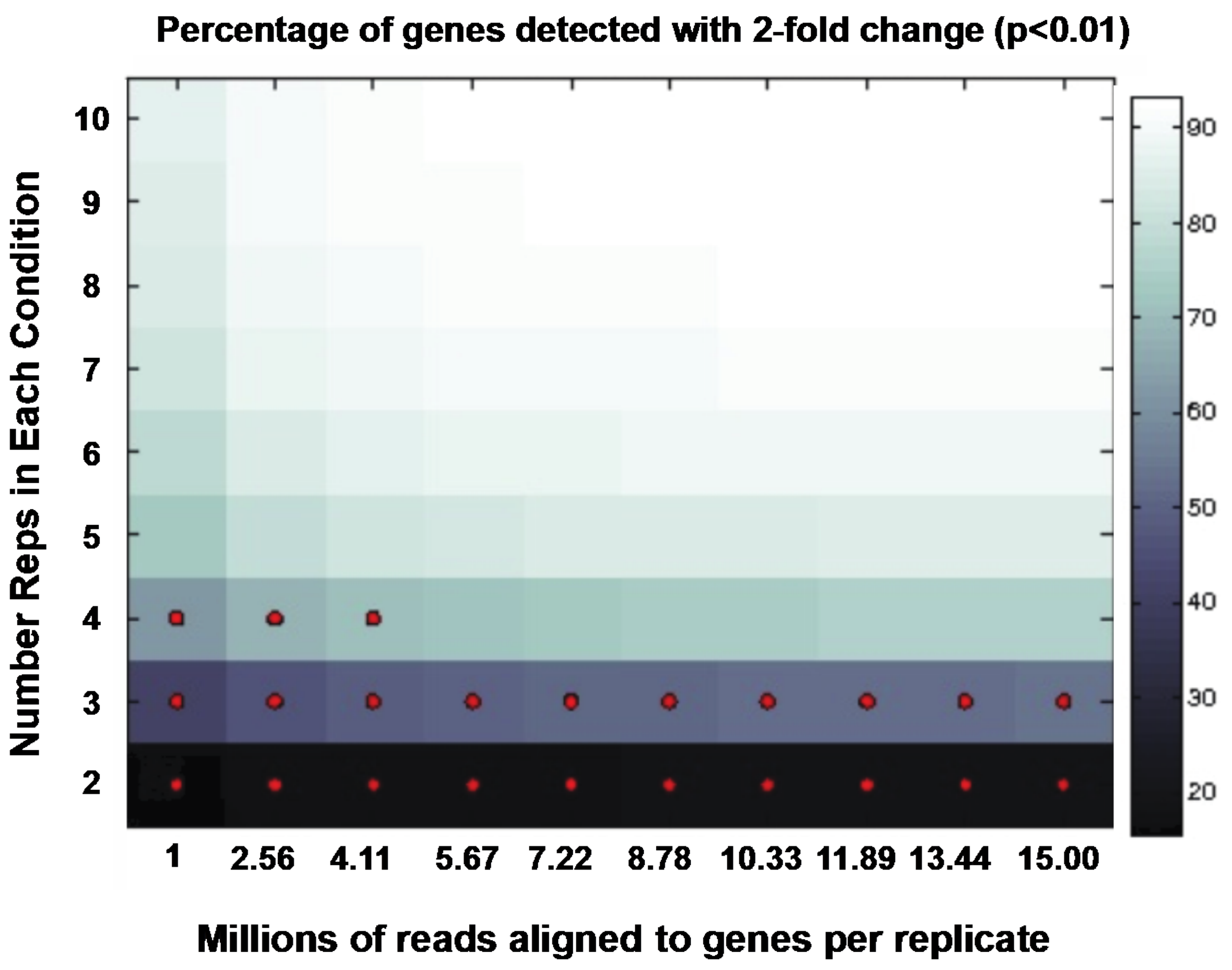

Figure S1. - Analysis of sequencing depth, number of biological replicates, and detection of differentially expressed genes (2-fold differential expression) using the online tool Scotty. For Scotty access instructions, please see https://github.com/amyschmid/rRNA_analysis/blob/main/scotty-access-instructions Squares with red dots are predicted to have <75% detection of differentially expressed genes.
